# Serum Folate and Plasma Homocysteine According to *MTHFR* C677T Genotype and Folic Acid Supplementation in Japanese Women Preparing for Pregnancy: A Cross-Sectional Study

**DOI:** 10.3390/nu18213594

**Published:** 2026-10-29

**Authors:** Fumitoshi Koga, Shogo Nakayama, Shigeki Kitagami, Masayo Matsunaga, Naoko Yokoyama, Tomoyuki Fujita

**Affiliations:** 1 Koga Fumitoshi Women’s Clinic, Fukuoka 810-0001, Japan; 2 Himawari Ladies Clinic Kokura, Fukuoka 802-0001, Japan

**Keywords:** folic acid, *MTHFR*, C677T polymorphism, homocysteine, one-carbon metabolism, preconception care, neural tube defects, precision nutrition

## Abstract

**Background:** Periconceptional folic acid supplementation reduces the risk of neural tube defects. Serum folate primarily reflects recent folate exposure, whereas plasma homocysteine may provide complementary, although non-specific, information on folate-related one-carbon metabolism. We investigated the relationships among serum folate, plasma homocysteine, methylenetetrahydrofolate reductase (*MTHFR*) C677T genotype, and folic acid supplementation in Japanese women preparing for pregnancy. **Methods:** This cross-sectional observational study included 359 Japanese women attending a fertility clinic who underwent *MTHFR* C677T genotyping and measurement of serum folate and plasma homocysteine. Participants were categorized according to reported supplemental folic acid intake (none, 400 µg/day, or 800 µg/day). Serum folate and plasma homocysteine concentrations were compared across supplementation categories within each genotype. Multivariable logistic regression was used to identify factors associated with plasma homocysteine concentrations ≥ 8.62 µmol/L, which was treated as an exploratory threshold rather than an established clinical cutoff. **Results:** Serum folate concentrations were significantly higher among women reporting folic acid supplementation across all three genotype groups. Plasma homocysteine concentrations differed across supplementation categories in the CT and TT genotype groups, but not in the CC genotype group. Among women with the TT genotype, the proportions with plasma homocysteine concentrations ≥ 8.62 µmol/L were 64.3% without supplementation, 25.0% with 400 µg/day, and 12.5% with 800 µg/day. In multivariable analysis, the TT genotype was associated with higher odds of plasma homocysteine ≥ 8.62 µmol/L compared with the CC genotype (adjusted OR, 2.65; 95% CI, 1.25–5.56), whereas reported supplementation with 400 µg/day and 800 µg/day was associated with lower odds compared with no supplementation (adjusted OR, 0.36; 95% CI, 0.19–0.67 and adjusted OR, 0.33; 95% CI, 0.16–0.65, respectively). **Conclusions:** Serum folate and plasma homocysteine provided complementary information across *MTHFR* C677T genotypes in this cohort. Plasma homocysteine should be interpreted in the context of multiple nutritional and genetic determinants and should not be regarded as a specific marker of folate status alone. Combined assessment of serum folate, plasma homocysteine, and *MTHFR* genotype may provide additional information for preconception nutritional evaluation. Further prospective studies are needed to determine the clinical utility of this approach.

## 1. Introduction

Neural tube defects (NTDs) are among the most common congenital malformations worldwide and arise from failure of neural tube closure during early embryogenesis [1].

Large randomized trials and population-based studies have demonstrated that periconceptional folic acid supplementation substantially reduces the incidence of neural tube defects (NTDs), leading many countries and international organizations to recommend daily folic acid supplementation for women planning pregnancy [2,3,4,5]. In Japan, women intending to conceive are advised to consume an additional 400 μg/day of folic acid in addition to dietary folate intake [6].

The methylenetetrahydrofolate reductase (MTHFR) enzyme plays a pivotal role in folate metabolism by catalyzing the conversion of 5,10-methylenetetrahydrofolate to 5-methyltetrahydrofolate, the methyl donor required for remethylation of homocysteine to methionine. The common C677T polymorphism reduces MTHFR enzymatic activity, particularly in individuals with the TT genotype, resulting in impaired methylation capacity and elevated plasma homocysteine concentrations despite apparently adequate folate concentrations [7,8,9].

Previous studies have shown that the *MTHFR* 677TT genotype is relatively common among Japanese women and may influence folate metabolism during the periconceptional period. Kuroda et al. reported that approximately two-thirds of Japanese women undergoing infertility treatment carried the CT or TT genotype and demonstrated improvements in serum folate and plasma homocysteine following multivitamin supplementation containing 800 µg of folic acid [10].

Serum folate concentration is widely used as an indicator of folate nutritional status because it reflects recent folate intake. However, serum folate does not necessarily indicate whether intracellular one-carbon metabolism is functioning adequately. Plasma total homocysteine may provide complementary information on folate-dependent one-carbon metabolism, although it is influenced by multiple nutritional and physiological factors [11,12,13].

Although folic acid supplementation effectively increases serum folate concentrations, it remains unclear whether conventional serum folate measurements alone sufficiently evaluate functional folate status in women with different *MTHFR* genotypes. We hypothesized that combining serum folate with plasma homocysteine would provide a complementary assessment of folate metabolism.

Therefore, the present study investigated the relationships among *MTHFR* C677T genotype, serum folate concentration, plasma homocysteine concentration, and folic acid supplementation in Japanese women attending a fertility clinic. To our knowledge, few studies have concurrently evaluated serum folate, plasma homocysteine, folic acid supplementation, and *MTHFR* C677T genotype in Japanese women preparing for pregnancy.

## 2. Materials and Methods

### 2.1. Study Design and Participants

This single-center observational study was conducted at Koga Fumitoshi Women’s Clinic (Fukuoka, Japan) between April and October 2023. Women aged ≥20 and <50 years who visited the clinic for fertility evaluation or treatment were eligible for inclusion if they were not pregnant at the time of evaluation, had provided written informed consent, and underwent measurement of serum folate, plasma total homocysteine, and *MTHFR* C677T genotyping. Participants with incomplete laboratory data or missing information regarding folic acid supplementation were excluded. A total of 359 women were included in the final analysis.

### 2.2. Assessment of Folic Acid Supplementation

Information regarding folic acid supplementation was obtained using a standardized questionnaire completed at the time of blood sampling.

Participants were classified into three groups according to their daily supplemental folic acid intake:

- No folic acid supplementation;
- 400 µg/day;
- 800 µg/day.

Daily folic acid intake refers to supplemental folic acid rather than total dietary folate equivalents. Dietary folate intake was not quantitatively assessed and therefore was not included in the classification.

### 2.3. MTHFR C677T Genotyping

Genotyping of the *MTHFR* C677T polymorphism was performed using the FolliCheck^®^ assay (Healthcare Systems Co., Ltd., Nagoya, Japan). Genomic DNA was extracted from buccal mucosal specimens collected from each participant.

The *MTHFR* C677T polymorphism (rs1801133) was analyzed using a TaqMan^®^ SNP Genotyping Assay (Assay ID: C__1202883_20; Applied Biosystems, Foster City, CA, USA) with THUNDERBIRD^®^ Probe qPCR Mix (TOYOBO Co., Ltd., Osaka, Japan) on a real-time polymerase chain reaction system according to the manufacturers’ instructions. PCR amplification was performed with an initial denaturation at 95 °C for 60 s, followed by 40 cycles of 95 °C for 15 s and 60 °C for 45 s. Genotypes were determined by allelic discrimination analysis and classified as CC, CT, or TT.

### 2.4. Measurement of Serum Folate and Plasma Homocysteine

Serum folate and plasma total homocysteine concentrations were measured at BML Inc. (Tokyo, Japan).

Serum folate concentrations were determined using a chemiluminescent immunoassay (CLIA) and expressed as ng/mL.

To minimize pre-analytical variation, blood samples for plasma homocysteine measurement were centrifuged promptly after collection, and the separated plasma was refrigerated until analysis. Plasma total homocysteine concentrations were measured using high-performance liquid chromatography and expressed as µmol/L. Throughout this manuscript, the term “plasma homocysteine” refers to plasma total homocysteine. Based on previous studies, a serum folate concentration of ≥7.0 ng/mL was used as a reference value related to neural tube risk reduction [11]. For plasma homocysteine, 8.62 µmol/L was used as an exploratory threshold based on the mean concentration reported by Mills et al. among mothers of infants with neural tube defects [14]. This value was not regarded as an established clinical cutoff.

### 2.5. Statistical Analysis

Continuous variables were summarized as means ± standard deviations or medians with interquartile ranges, depending on their distributions. Categorical variables were presented as numbers and percentages.

Differences among the CC, CT, and TT genotype groups were analyzed using the Kruskal–Wallis test for continuous variables and the chi-square test or Fisher’s exact test for categorical variables, as appropriate.

Hardy–Weinberg equilibrium was evaluated using an exact test.

Within each genotype group, serum folate and plasma homocysteine concentrations were compared among participants receiving no supplemental folic acid, 400 µg/day, or 800 µg/day. When overall differences were significant, post hoc pairwise comparisons were performed using Dunn’s test with Bonferroni correction.

The proportions of participants with plasma homocysteine concentrations ≥ 8.62 µmol/L were compared according to *MTHFR* genotype and folic acid supplementation category.

Multivariable logistic regression analysis was performed to identify factors independently associated with plasma homocysteine concentrations ≥ 8.62 µmol/L. The explanatory variables included *MTHFR* C677T genotype, folic acid supplementation category, age, and body mass index (BMI). Age and BMI were selected a priori as covariates because they are established or plausible determinants of plasma homocysteine concentrations and were available for all participants. The CC genotype and no folic acid supplementation were used as the reference categories. Adjusted odds ratios (ORs) and 95% confidence intervals (CIs) were calculated.

To formally evaluate whether the association between folic acid supplementation and plasma homocysteine differed according to *MTHFR* genotype, plasma homocysteine concentrations were logarithmically transformed because of their right-skewed distribution and analyzed using two-way analysis of variance within a general linear model framework, with *MTHFR* genotype, folic acid supplementation category, and their interaction term included in the model. The genotype × folic acid supplementation interaction was also evaluated in logistic regression models using plasma homocysteine ≥ 8.62 µmol/L as a binary outcome. As a sensitivity analysis, the logistic regression analysis was repeated using an alternative cutoff of 10.0 µmol/L. Statistical analyses were performed using R (version 4.6.0) and RStudio (version 2026.05.0+218).

### 2.6. Ethical Considerations

The study protocol was approved by the Ethics Committee of Koga Fumitoshi Women’s Clinic (Approval No. 2023-2; approval date: 22 March 2023). Written informed consent was obtained from all participants before enrollment. The study was conducted in accordance with the Declaration of Helsinki.

## 3. Results

### 3.1. Participant Characteristics

A total of 359 women were included in the final analysis. The mean age of the participants was 38.3 ± 4.7 years, the mean body mass index (BMI) was 21.5 ± 3.4 kg/m^2^, and the mean duration of infertility was 4.4 ± 3.4 years (Table 1).

The distribution of *MTHFR* C677T genotypes was 135 (37.6%) participants with the CC genotype, 174 (48.5%) with the CT genotype, and 50 (13.9%) with the TT genotype. Overall, 224 participants (62.4%) carried at least one T allele (CT or TT) (Table 2).

Regarding supplemental folic acid intake, 86 women received no supplementation, 161 received 400 µg/day, and 112 received 800 µg/day. Within the CC genotype, the corresponding numbers were 28, 59, and 48; within the CT genotype, 44, 82, and 48; and within the TT genotype, 14, 20, and 16, respectively (Table 1).

No significant differences were observed among the three genotype groups with respect to age, BMI, duration of infertility, infertility type, smoking status, or supplemental folic acid category (all *p* > 0.05). The genotype distribution was consistent with Hardy–Weinberg equilibrium (*p* = 0.656).

### 3.2. Serum Folate Concentrations According to MTHFR Genotype and Folic Acid Supplementation

Serum folate concentrations were significantly higher among women reporting folic acid supplementation in all three genotype groups (all overall *p* < 0.001) (Table 3).

Among participants with the CC genotype, the median serum folate concentrations were 9.05 ng/mL in the non-supplemented group, 17.70 ng/mL in the 400 µg/day group, and 25.90 ng/mL in the 800 µg/day group. The corresponding values were 8.45, 18.15, and 20.65 ng/mL in participants with the CT genotype, and 7.50, 16.10, and 18.10 ng/mL in those with the TT genotype, respectively.

Pairwise comparisons showed significantly higher serum folate concentrations in both supplementation groups than in the non-supplemented group for all three genotypes (all *p* < 0.001). No significant differences were observed between the 400 µg/day and 800 µg/day groups within any genotype.

### 3.3. Plasma Homocysteine Concentrations According to MTHFR Genotype and Folic Acid Supplementation

Plasma homocysteine concentrations differed across folic acid supplementation categories in the CT and TT genotype groups, whereas no significant difference was observed in the CC genotype group (Table 3).

Among participants with the CC genotype, the median plasma homocysteine concentrations were 7.00 µmol/L in the non-supplemented group, 6.50 µmol/L in the 400 µg/day group, and 6.30 µmol/L in the 800 µg/day group, with no significant overall difference among the three supplementation categories (overall *p* = 0.308).

Among participants with the CT genotype, the corresponding median plasma homocysteine concentrations were 7.65, 6.80, and 6.10 µmol/L, respectively (overall *p* < 0.001). Pairwise comparisons showed significantly lower plasma homocysteine concentrations in both the 400 µg/day group (*p* = 0.004) and the 800 µg/day group (*p* < 0.001) compared with the non-supplemented group, whereas the difference between the 400 µg/day and 800 µg/day groups was not statistically significant (*p* = 0.066).

Among participants with the TT genotype, the corresponding median plasma homocysteine concentrations were 9.20, 6.80, and 6.50 µmol/L, respectively (overall *p* = 0.002). Pairwise comparisons showed significantly lower plasma homocysteine concentrations in both the 400 µg/day group (*p* = 0.017) and the 800 µg/day group (*p* = 0.002) compared with the non-supplemented group, with no significant difference between the two supplementation groups (*p* = 1.000).

When plasma homocysteine was analyzed as a continuous variable after logarithmic transformation, a significant genotype × folic acid supplementation interaction was observed (*p* for interaction = 0.038).

### 3.4. Participants with Plasma Homocysteine Concentrations ≥ 8.62 µmol/L

The proportion of participants with plasma homocysteine concentrations ≥ 8.62 µmol/L was compared across folic acid supplementation categories within each *MTHFR* genotype group (Figure 1).

Among participants with the CC genotype, the proportions were 28.6% (8/28) in the non-supplemented group, 11.9% (7/59) in the 400 µg/day group, and 14.6% (7/48) in the 800 µg/day group, with no significant overall difference among supplementation categories (*p* = 0.132). Among participants with the CT genotype, the corresponding proportions were 25.0% (11/44), 14.6% (12/82), and 10.4% (5/48), respectively, again with no significant overall difference (*p* = 0.145).

In contrast, among participants with the TT genotype, the proportions were 64.3% (9/14) in the non-supplemented group, 25.0% (5/20) in the 400 µg/day group, and 12.5% (2/16) in the 800 µg/day group, with a significant overall difference across supplementation categories (*p* = 0.0069). In post hoc pairwise comparisons with Bonferroni correction, the difference between the non-supplemented and 800 µg/day groups remained statistically significant (adjusted *p* = 0.020), whereas the differences between the non-supplemented and 400 µg/day groups (adjusted *p* = 0.105) and between the 400 and 800 µg/day groups (adjusted *p* = 1.000) were not statistically significant.

These findings indicate that, using the exploratory threshold of 8.62 µmol/L, the proportion of participants with elevated plasma homocysteine differed across supplementation categories in the TT genotype group, whereas no significant differences were observed in the CC or CT genotype groups.

### 3.5. Multivariable Logistic Regression Analysis of Factors Associated with Plasma Homocysteine Concentrations ≥ 8.62 µmol/L

Multivariable logistic regression analysis was performed to identify factors associated with plasma homocysteine concentrations ≥ 8.62 µmol/L (Table 4).

Compared with the CC genotype, the TT genotype was independently associated with higher odds of elevated plasma homocysteine (adjusted OR, 2.65; 95% CI, 1.25–5.56; *p* = 0.010), whereas the CT genotype was not significantly associated with elevated homocysteine (adjusted OR, 1.11; 95% CI, 0.61–2.05; *p* = 0.725).

Both folic acid supplementation groups were independently associated with lower odds of elevated plasma homocysteine. Compared with no supplementation, supplementation with 400 µg/day and 800 µg/day was associated with adjusted ORs of 0.36 (95% CI, 0.19–0.67; *p* = 0.001) and 0.33 (95% CI, 0.16–0.65; *p* = 0.002), respectively.

A formal test for genotype × folic acid supplementation interaction showed no significant interaction when elevated plasma homocysteine was defined using the exploratory cutoff of 8.62 µmol/L (likelihood-ratio *p* = 0.479).

In a sensitivity analysis using a higher cutoff of 10.0 µmol/L, the genotype × folic acid supplementation interaction likewise remained non-significant (likelihood-ratio *p* = 0.658). In the model without the interaction term, both 400 µg/day and 800 µg/day supplementation were associated with lower odds of plasma homocysteine ≥ 10.0 µmol/L compared with no supplementation (OR, 0.28; 95% CI, 0.12–0.62; *p* = 0.002 and OR, 0.36; 95% CI, 0.15–0.82; *p* = 0.017, respectively).

## 4. Discussion

This study examined the relationships among serum folate concentration, plasma homocysteine concentration, folic acid supplementation, and the *MTHFR* C677T genotype in Japanese women preparing for pregnancy. Serum folate concentrations were higher among women reporting folic acid supplementation across all three genotype groups. Plasma homocysteine concentrations differed across supplementation categories in the CT and TT genotype groups, whereas no significant difference was observed in the CC genotype group. These findings suggest that serum folate and plasma homocysteine may provide complementary information when interpreted in the context of *MTHFR* genotype.

The observed genotype-related differences are biologically plausible given the established role of *MTHFR* in one-carbon metabolism. MTHFR catalyzes the conversion of 5,10-methylenetetrahydrofolate to 5-methyltetrahydrofolate, which provides the methyl group required for the remethylation of homocysteine to methionine [7,15]. Experimental studies have also suggested that adequate methionine availability is important for normal neural tube closure, further supporting the biological relevance of one-carbon metabolism during early embryonic development [16]. The C677T variant reduces MTHFR enzymatic activity, particularly in individuals with the TT genotype, and has been associated with differences in folate and homocysteine metabolism [7,8,9,10,17,18]. However, plasma homocysteine is influenced by multiple nutritional, genetic, and physiological factors and should not be interpreted as a specific marker of folate status alone [12,13,15]. The core folate-dependent remethylation pathway and selected nutritional cofactors relevant to homocysteine metabolism are summarized schematically in Figure 2.

Our findings are broadly consistent with previous studies showing differences in serum folate and plasma homocysteine according to folic acid supplementation and *MTHFR* C677T genotype. Kuroda et al. reported that supplementation with a multivitamin containing 800 µg of folic acid increased serum folate and decreased plasma homocysteine concentrations in Japanese women undergoing infertility treatment [10]. Miyaki et al. also demonstrated genotype-related differences in the homocysteine response to folic acid supplementation [17]. In the present observational study, women reporting folic acid supplementation had higher serum folate concentrations, while plasma homocysteine concentrations differed across supplementation categories in the CT and TT genotype groups. Unlike these interventional studies, however, the present observational analysis cannot establish that folic acid supplementation itself caused the observed differences.

An important consideration is that participants in the present study were classified according to supplemental folic acid intake rather than total folate intake. Dietary folate intake was not quantitatively assessed, and detailed information on supplement composition was not available for all participants. Therefore, differences in plasma homocysteine cannot be attributed to folic acid dose alone. Homocysteine metabolism also depends on nutritional cofactors including vitamin B12, vitamin B6, and riboflavin [12,13,15,19]. Other nutritional and physiological factors may also contribute to interindividual variation in plasma homocysteine [18,19,20,21,22,23,24]. Residual confounding by these factors therefore cannot be excluded.

Accordingly, the present findings should be interpreted as indicating that serum folate and plasma homocysteine provide complementary, but not interchangeable, information in preconception nutritional assessment. Serum folate primarily reflects recent folate exposure and circulating folate availability, whereas plasma homocysteine reflects the integrated activity of one-carbon metabolism and is influenced by several nutritional and non-nutritional determinants [7,12,13,15]. Thus, an apparently adequate serum folate concentration does not necessarily imply a low plasma homocysteine concentration, particularly across different *MTHFR* genotypes. Conversely, elevated plasma homocysteine should not be regarded as evidence of folate insufficiency in isolation.

The plasma homocysteine threshold of 8.62 µmol/L used in the present study was selected for exploratory purposes based on the mean concentration reported by Mills et al. among mothers of infants with neural tube defects (8.62 ± 2.8 µmol/L), which was significantly higher than that in matched controls (7.96 ± 2.5 µmol/L) [14]. Subsequent studies have likewise reported higher maternal homocysteine concentrations in pregnancies affected by neural tube defects, although the absolute concentrations have varied across study populations. Zhao et al. reported concentrations of 9.40 µmol/L in women with NTD-affected pregnancies compared with 7.56 µmol/L in controls [25], whereas Gu et al. reported values of 15.1 ± 7.8 and 8.5 ± 4.0 µmol/L, respectively [26]. In addition, meta-analyses published in 2015 and 2016 confirmed that maternal homocysteine concentrations were significantly higher in NTD-affected pregnancies, but neither established a specific clinical threshold for NTD risk [27,28]. These differences across studies likely reflect heterogeneity in study populations, nutritional status, timing of blood sampling, and other determinants of homocysteine metabolism. Accordingly, 8.62 µmol/L should not be interpreted as a diagnostic cutoff, but rather as an exploratory threshold used to examine the present dataset. Accordingly, we additionally analyzed plasma homocysteine as a continuous variable and performed a sensitivity analysis using an alternative cutoff of 10.0 µmol/L.

## 5. Strengths, Limitations, and Future Perspectives

This study has several strengths. It concurrently evaluated serum folate, plasma homocysteine, folic acid supplementation, and *MTHFR* C677T genotype in Japanese women preparing for pregnancy. Previous Japanese studies have examined various relationships among *MTHFR* genotype, folate-related factors, and plasma homocysteine [10,17,18], but relatively few have assessed these variables concurrently in a preconception fertility-clinic population. This approach allowed the relationships among biochemical, nutritional, and genetic factors to be examined within the same clinical cohort.

Several limitations should also be acknowledged. First, this was a single-center observational study involving Japanese women attending a fertility clinic, which limits the generalizability of the findings to other populations. Second, folic acid supplementation was assessed by self-report, and dietary folate intake and adherence to supplementation were not quantitatively assessed. Third, detailed supplement composition, including vitamin B12, vitamin B6, and riboflavin content, was not available for all participants. These nutrients are relevant because they are involved in one-carbon and homocysteine metabolism [12,13,19]. Fourth, additional biomarkers related to one-carbon metabolism, including red blood cell folate and other nutritional cofactors, were not measured. Consequently, residual confounding by nutritional status, renal function, medications, lifestyle factors, and other determinants of plasma homocysteine cannot be excluded.

Another limitation is that the threshold of 8.62 µmol/L was used as an exploratory classification rather than as an established clinical cutoff [14]. Dichotomization of plasma homocysteine at a single threshold may also result in loss of information contained in its continuous distribution. To address this limitation, we additionally analyzed plasma homocysteine as a continuous variable and performed a sensitivity analysis using an alternative cutoff of 10.0 µmol/L to assess the robustness of the observed associations.

Overall, the present study suggests that serum folate and plasma homocysteine capture different aspects of folate-related and one-carbon metabolism and may provide complementary information when interpreted together with the *MTHFR* C677T genotype. However, plasma homocysteine is influenced by multiple nutritional and non-nutritional factors, and the present observational findings do not establish a clinical definition of functional folate sufficiency or demonstrate the benefit of genotype-guided intervention.

These findings may also be relevant to broader efforts to incorporate nutritional and metabolic assessment into preconception care, for which structured preconception check-up programs have recently been explored [29]. Prospective studies are needed to determine whether these measures are associated with reproductive and pregnancy outcomes [30,31] and interventional studies are required before genotype-guided or biomarker-guided supplementation strategies can be recommended.

## 6. Conclusions

Serum folate concentration and plasma homocysteine provided complementary information across *MTHFR* C677T genotypes in this cohort of Japanese women preparing for pregnancy. Elevated plasma homocysteine should be interpreted as a marker influenced by multiple nutritional and genetic factors rather than folate status alone. The combined assessment of serum folate, plasma homocysteine, and *MTHFR* genotype may provide complementary insight into preconception nutritional evaluation. Further prospective studies are needed to determine the clinical utility of this approach.

## Figures and Tables

**Figure 1:**
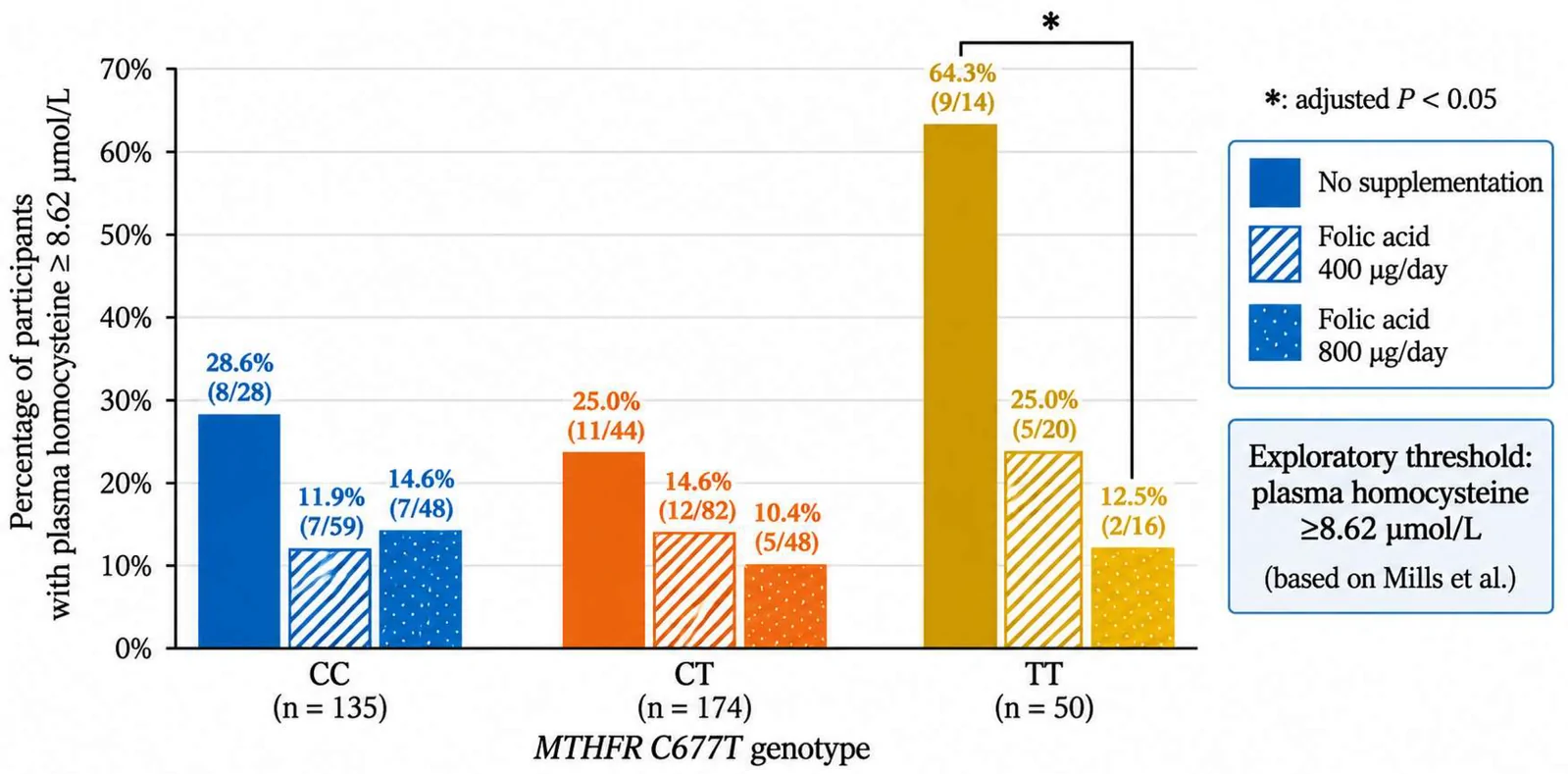
Percentage of participants with plasma homocysteine ≥ 8.62 µmol/L according to *MTHFR* C677T genotype and folic acid supplementation. Within-genotype χ^2^ tests showed *p* = 0.132 for CC, *p* = 0.145 for CT, and *p* = 0.0069 for TT. Post hoc Bonferroni-corrected comparisons in the TT genotype group showed adjusted *p* = 0.105 for no supplementation versus 400 µg/day, adjusted *p* = 0.020 for no supplementation versus 800 µg/day, and adjusted *p* = 1.000 for 400 versus 800 µg/day. Numbers above the bars indicate the percentage and *n*/N. * Adjusted *p* < 0.05 [14].

**Figure 2:**
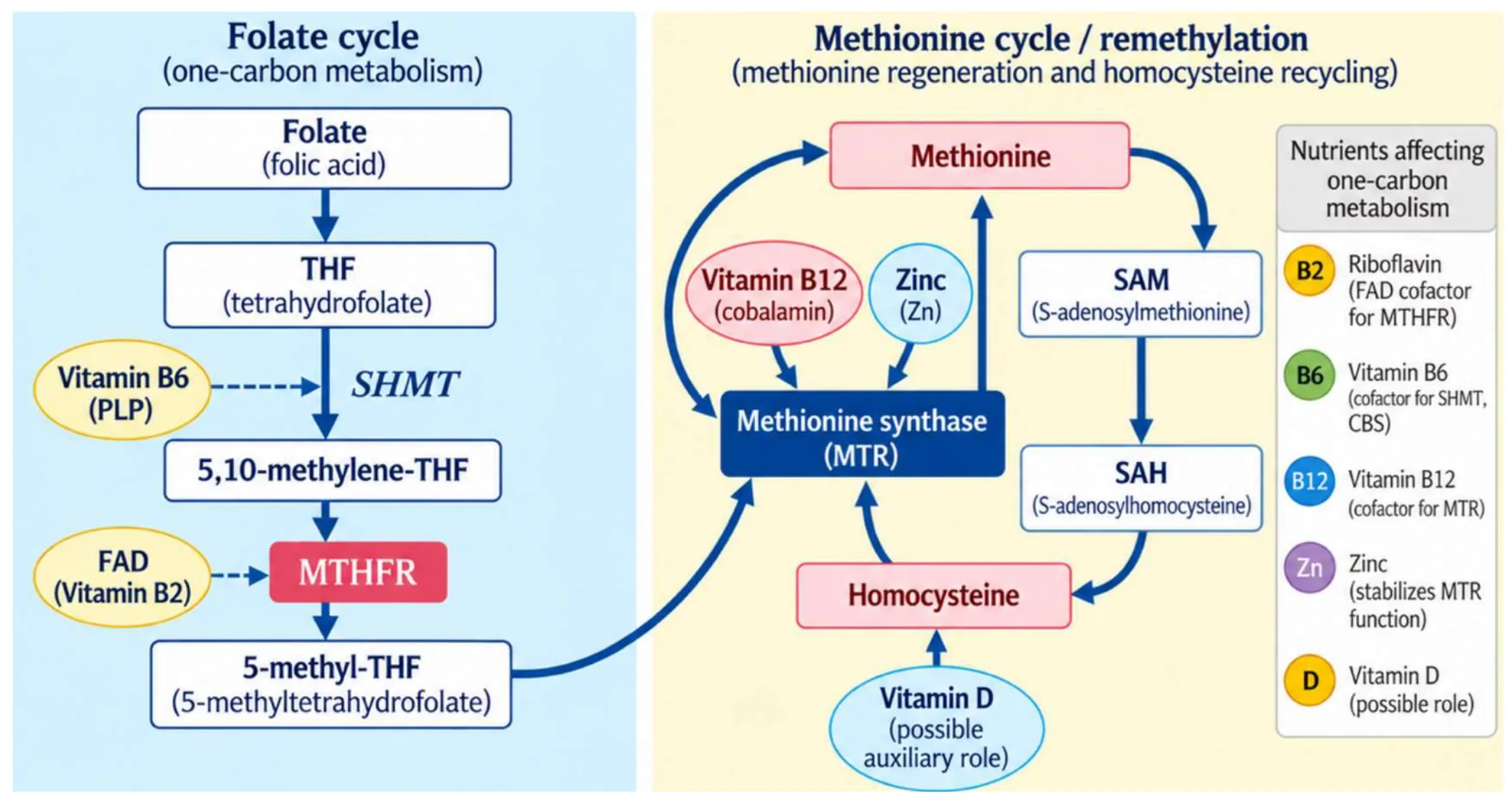
Schematic overview of folate-dependent homocysteine remethylation and related nutritional factors. Folate is converted to 5,10-methylene-THF, which is reduced by MTHFR to 5-methyl-THF. Methyl groups from 5-methyl-THF are used by methionine synthase (MTR), with vitamin B12 as a cofactor, to remethylate homocysteine to methionine. S-adenosylmethionine (SAM) serves as a universal methyl donor, and S-adenosylhomocysteine (SAH) is hydrolyzed to homocysteine. Nutritional factors, including vitamins B2, B6, and B12, zinc, and vitamin D, may influence this pathway and contribute to interindividual variability in homocysteine concentrations, particularly in individuals with the MTHFR C677T polymorphism. Original figure created by the authors. Generative AI was used solely to assist with the graphical rendering and layout of Figure 2. All scientific content, interpretation, labelling, and final verification were performed by the authors.

**Table 1:** Characteristics of the Study Participants.

Characteristic	Overall (*n* = 359)	CC (*n* = 135)	CT (*n* = 174)	TT (*n* = 50)	*p* Value
Age, years	38.3 (4.7)	38.1 (4.6)	38.4 (4.7)	38.9 (4.9)	0.359
BMI, kg/m^2^	21.5 (3.4)	21.7 (3.4)	21.4 (3.4)	21.4 (3.5)	0.620
Duration of infertility, years	4.4 (3.4)	4.1 (3.1)	4.4 (3.3)	5.3 (4.0)	0.395
Primary infertility, *n* (%)	182 (50.7)	75 (55.6)	87 (50.0)	20 (40.0)	0.165
Secondary infertility, *n* (%)	177 (49.3)	60 (44.4)	87 (50.0)	30 (60.0)	0.165
Current smokers, *n* (%)	17 (4.7)	9 (6.7)	6 (3.4)	2 (4.0)	0.400
Folic acid supplementation, *n* (%)	273 (76.0)	107 (79.3)	130 (74.7)	36 (72.0)	0.500
400 µg/day	161 (44.8)	59 (43.7)	82 (47.1)	20 (40.0)	0.630
800 µg/day	112 (31.2)	48 (35.6)	48 (27.6)	16 (32.0)	0.322
Hardy–Weinberg equilibrium	–	–	–	–	0.656

Data are presented as mean (standard deviation) or number (%), as appropriate. *p* values were calculated using the Kruskal–Wallis test for continuous variables and the χ^2^ test or Fisher’s exact test for categorical variables, as appropriate. The genotype distribution did not deviate significantly from Hardy–Weinberg equilibrium (exact test, *p* = 0.656). Folic acid supplementation included supplements providing either 400 or 800 µg/day of folic acid. Abbreviations: BMI, body mass index; CC, wild-type homozygote; CT, heterozygote; TT, variant homozygote.

**Table 2:** Distribution of *MTHFR* C677T Genotypes.

Genotype	*n*	%
CC	135	37.6
CT	174	48.5
TT	50	13.9
CT/TT (carriers of T allele)	224	62.4
Total	359	100.0

CT/TT represents participants carrying at least one T allele (CT or TT genotype).

**Table 3:** Serum folate concentrations and plasma homocysteine levels according to *MTHFR* C677T genotype and folic acid supplementation.

Variable	Genotype	No Supplementation	400 µg/Day	800 µg/Day	*p* Value †	No Suppl. vs. 400	No Suppl. vs. 800	400 vs. 800 ‡
Serum folate (ng/mL)	CC (*n* = 135)	9.05 (7.22–12.50) *n* = 28	17.70 (13.50–22.90) *n* = 59	25.90 (14.8–35.6) *n* = 48	<0.001	<0.001	<0.001	0.054
	CT (*n* = 174)	8.45 (6.68–10.5) *n* = 44	18.15 (12.0–23.4) *n* = 82	20.65 (15.6–29.9) *n* = 48	<0.001	<0.001	<0.001	0.253
	TT (*n* = 50)	7.50 (4.93–9.18) *n* = 14	16.10 (11.7–22.3) *n* = 20	18.10 (14.4–26.8) *n* = 16	<0.001	<0.001	<0.001	1.000
Homocysteine (µmol/L)	CC (*n* = 135)	7.00 (6.00–8.95) *n* = 28	6.50 (5.80–7.30) *n* = 59	6.30 (5.60–8.12) *n* = 48	0.308	0.616	0.413	1.000
	CT (*n* = 174)	7.65 (6.98–8.67) *n* = 44	6.80 (5.93–7.60) *n* = 82	6.10 (5.50–6.90) *n* = 48	<0.001	0.004	<0.001	0.066
	TT (*n* = 50)	9.20 (7.70–10.68) *n* = 14	6.80 (6.20–8.48) *n* = 20	6.50 (5.57–8.02) *n* = 16	0.002	0.017	0.002	1.000

Data are presented as median (interquartile range). † Overall comparisons among the three supplementation groups were performed using the Kruskal–Wallis test. ‡ Pairwise comparisons were performed using Dunn’s post hoc test with Bonferroni correction.

**Table 4:** Multivariable logistic regression analysis for factors associated with elevated plasma homocysteine (≥8.62 µmol/L).

Variable	Odds Ratio (OR)	95% Confidence Interval (CI)	*p* Value
CT genotype vs. CC genotype	1.11	0.614–2.047	0.725
TT genotype vs. CC genotype	2.65	1.25–5.56	0.010
Folic acid supplementation (400 µg/day vs. No supplementation)	0.361	0.194–0.669	0.001
Folic acid supplementation (800 µg/day vs. No supplementation)	0.328	0.161–0.647	0.002
Age (years)	0.985	0.932–1.042	0.589
BMI (kg/m^2^)	1.031	0.955–1.107	0.418

Adjusted for age and BMI. Abbreviations: BMI, body mass index; CC, wild-type homozygote; CT, heterozygote; TT, variant homozygote; OR, odds ratio; CI, confidence interval.

## Data Availability

The data presented in this study are available from the corresponding author upon reasonable request. The data are not publicly available because they contain information that could compromise the privacy of the research participants.
